# Nutritional support during the first week for infants with bronchopulmonary dysplasia and respiratory distress: a multicenter cohort study in China

**DOI:** 10.1186/s12887-024-04675-5

**Published:** 2024-04-03

**Authors:** Huijia Lin, Guannan Bai, Jiajing Ge, Xuefeng Chen, Xinyu He, Xiaolu Ma, Liping Shi, Lizhong Du, Zheng Chen

**Affiliations:** 1 Department of NICU, Children’s Hospital, Zhejiang University School of Medicine, National Clinical Research Center for Child Health, Hangzhou, China; 2 Department of Child Health Care, Children’s Hospital, Zhejiang University School of Medicine, National Clinical Research Center for Child Health, Hangzhou, China; 3 Department of Endocrinology, Children’s Hospital, Zhejiang University School of Medicine, National Clinical Research Center for Child Health, Hangzhou, China

**Keywords:** Bronchopulmonary dysplasia, Nutrition, Preterm infant, Breastmilk

## Abstract

**Background:**

Bronchopulmonary dysplasia (BPD) is a major complication affecting the survival rate and long-term outcomes of preterm infants. A large, prospective, multicenter cohort study was conducted to evaluate early nutritional support during the first week of life for preterm infants with a gestational age < 32 weeks and to verify nutritional risk factors related to BPD development.

**Methods:**

A prospective multicenter cohort study of very preterm infants was conducted in 40 tertiary neonatal intensive care units across mainland China between January 1, 2020, and December 31, 2021. Preterm infants who were born at a gestational age < 32 weeks, < 72 h after birth and had a respiratory score > 4 were enrolled. Antenatal and postnatal information focusing on nutritional parameters was collected through medical systems. Statistical analyses were also performed to identify BPD risk factors.

**Results:**

The primary outcomes were BPD and severity at 36 weeks postmenstrual age. A total of 1410 preterm infants were enrolled in this study. After applying the exclusion criteria, the remaining 1286 infants were included in this analysis; 614 (47.7%) infants were in the BPD group, and 672 (52.3%) were in the non-BPD group. In multivariate logistic regression model, the following six factors were identified of BPD: birth weight (OR 0.99, 95% CI 0.99–0.99; *p* = 0.039), day of full enteral nutrition (OR 1.03, 95% CI 1.02–1.04; *p* < 0.001), parenteral protein > 3.5 g/kg/d during the first week (OR 1.65, 95% CI 1.25–2.17; *p* < 0.001), feeding type (formula: OR 3.48, 95% CI 2.21–5.49; *p* < 0.001, mixed feed: OR 1.92, 95% CI 1.36–2.70; *p* < 0.001; breast milk as reference), hsPDA (OR 1.98, 95% CI 1.44–2.73; *p* < 0.001), and EUGR ats 36 weeks (OR 1.40, 95% CI 1.02–1.91; *p* = 0.035).

**Conclusions:**

A longer duration to achieve full enteral nutrition in very preterm infants was associated with increased BPD development. Breastfeeding was demonstrated to have a protective effect against BPD. Early and rapidly progressive enteral nutrition and breastfeeding should be promoted in very preterm infants.

**Trial registration:**

The trial was registered in the Chinese Clinical Trial Registry (No. ChiCTR2000030125 on 24/02/2020) and in www.ncrcch.org (No. ISRCTN84167642 on 25/02/2020).

**Supplementary Information:**

The online version contains supplementary material available at 10.1186/s12887-024-04675-5.

## Background

Due to advancements in perinatal and neonatal care in China, more preterm newborns are receiving treatment and surviving. However, the lung development of preterm infants is susceptible to various injuries, resulting in bronchopulmonary dysplasia (BPD) [1]. The prevalence of BPD in China has risen over the last several decades [2, 3].

BPD is one of the most prevalent and complicated infant sequelae and places a significant burden on newborns, families, and society. Moderate/severe BPD is associated with short- and long-term serious growth restrictions, impaired cardiorespiratory function, and neurodevelopmental disability [4]. The etiology of BPD is multifactorial and is frequently associated with several genetic, prenatal, and postnatal variables modulating lung development and the response to local and systemic inflammation.

Nutrition is crucial for infants with BPD, as sick newborns expend large amounts of energy to compensate for their high metabolic demands and increased work in breathing [5]. Additionally, malnutrition impacts lung development, and early inadequate caloric intake during life is associated with the development of BPD [6].

Therefore, we conducted a multicenter cohort study to evaluate the risk factors for nutritional status during the first week after the progression of BPD in preterm infants with a gestational age (GA) < 32 weeks, < 72 h after birth and a respiratory score > 4.

## Methods

### Study design and patients

We performed a prospective multicenter cohort study from January 1, 2020, to December 31, 2021. Forty tertiary referral neonatal intensive care units (NICUs) from 21 provinces and cities across mainland China participated in this observational study. Preterm infants with a GA < 32 weeks, < 72 h after birth and a respiratory distress score > 4 at admission were enrolled. This score, evaluated according to the Acute Care of At-Risk Newborns Respiratory Distress Score System, was the sum of the 6 individual scores and is useful for tracking the severity of respiratory distress over time in a baby who is breathing spontaneously (Table 1) [7]. The respiratory score was collapsed into three levels: mild, 0 to 4; moderate, 5 to 8; and severe, > 8 or intubated at admission.

**Table 1:** Acute Care of at-Risk Newborns Respiratory Distress Score System

Score	0	1	2
Respiratory rate	40—60/mins	60—80/mins	> 80/mins
Oxygen requirement	None	≤ 50%	> 50%
Retractions	None	Mild to moderate	Severe
Grunting	None	With stimulation	Continuous at rest
Breath sounds on auscultation	Easily heard throughout	Decreased	Barely heard
Prematurity	> 34 wks	30—34 wks	< 30 wks

Wks, weeks

A brief diagnosis can be obtained during physical examination at admission, when there is no blood test requirement, and when there is a greater focus on respiratory diseases [8]. Furthermore, we observed that preterm infants whose scores were less than 4 throughout the first 72 h of life were nearly always normal and rarely progressed to BPD from a clinical standpoint. Therefore, we hypothesized that nutritional support during the first week was associated with the progression of BPD in very preterm infants with a score greater than 4.

The exclusion criteria were major congenital anomalies, including serious congenital heart defects, chromosomal abnormalities, brain malformations, congenital diaphragmatic hernias, digestive tract or kidney anomalies and inborn errors of metabolism or severe heritable disease.

The dropout criterion included death within 14 days after birth or discontinuation of treatment within 36 weeks postmenstrual age (PMA).

The study was approved by the Children Hospital Ethics Committee, Zhejiang University School of Medicine Ethical (2019-IRB-164) on December 24, 2019. Written informed consent was obtained from parents or guardians antenatally or upon NICU admission. The trial was registered in the Chinese Clinical Trial Registry (No. ChiCTR2000030125 on 24/02/2020) and at www.ncrcch.org (No. ISRCTN84167642 on 25/02/2020).

### Definitions of the relevant concepts

BPD was diagnosed and graded according to the 2018 National Institute of Child Health and Human Development (NICHD) consensus [9]. Extrauterine growth retardation (EUGR) was described according to the Fenton intrauterine growth curves as infants whose weight was less than the 10th percentile for corrected gestational age [10]. Intrauterine growth retardation (IUGR) was defined as a birth weight below the 10th percentile for gestational age [11]. Hemodynamically significant patent ductus arteriosus (hsPDA) was characterized by a ductus diameter exceeding 1.5 mm, a left atrial inner diameter/aortic root (LA/AO) exceeding 1.4, or a combined left-to-right shunt, as determined by echocardiography. Necrotizing enterocolitis (NEC) was diagnosed based on the modified Bell staging system, clinical features, and radiological evidence with a Bell stage greater than 2 [12].

### Data collection

Maternal, perinatal, and neonatal data were obtained from family interviews and from the medical charts or electronic systems of the enrolled infants. Antenatal and neonatal characteristics, including gestational age (GA) at delivery and birth weight (BW), sex, delivery mode, 5-minute Apgar score, intrauterine growth retardation, pregnancy-induced hypertension, maternal diabetes, and premature rupture of membranes, were collected. The following neonatal outcomes and morbidity data were also recorded: respiratory distress scores, invasive mechanical ventilation duration, surfactant replacement, necrotizing enterocolitis, hsPDA, prognosis, EUGR at 36 weeks, and BPD.

Our study focused on nutritional strategies and their related outcomes. Therefore, nutritional data were also collected prospectively, including whether the total fluid intake was > 150 ml/kg/d during the first week, whether the parenteral amino acid intake was > 3.5 g/kg/d, or whether the total energy intake was > 100 kcal/kg/d. Additionally, weight change, the day weight recovered to the birth weight, and when weight loss after birth exceeded 10% birth weight were recorded. In addition to parenteral nutrition (PN), enteral nutrition (EN) is crucial for preterm infants. We focused on the day that the total EN was reached and the feeding type, including exclusive breastmilk, formula, breastmilk > 50%, or exclusive feeding. Additionally, EUGR data at 36 weeks were acquired.

All infants were divided into two groups according to whether their parenteral AA concentration was > 3.5 g/kg/d during the first week compared with the days at which full enteral nutrition was achieved. Additionally, infants were divided into subgroups according to the day at which full EN was reached: 14–28 days, 28–42 days, and > 42 days, compared with the rate of parenteral AA > 3.5 g/kg/d.

All the data were entered into the Resman research manager database of the ChiCTR for subsequent analysis. Only study members had permission to access the database, and a specialist kept all the data to protect patient privacy.

### Outcomes

To accomplish an analysis according to the principle of intention to identify risk factors, we defined our primary outcome as BPD at 36 weeks postmenstrual age (PMA). Participants were assessed at 36 ± 1 weeks’ PMA and BPD severity, classified as the 2018 National Institutes of Health Group (NICHD) Workshop, based on modes of respiratory support grades [8]. The secondary outcomes were complications and risk factors for nutritional support during the first week associated with BPD occurrence.

### Statistical analysis

First, we conducted a descriptive analysis. For continuous variables, normality was checked. Variables without a normal distribution are presented as medians (interquartile ranges [IQRs]). Categorical variables are presented as numbers and percentages. Second, for the continuous variables, we applied one-way ANOVA to compare the differences across subgroups, and for the categorical variables, we used chi-square tests to compare the differences across subgroups. To further clarify the difference between two groups, we conducted post hoc analysis via one-way ANOVA and chi-square tests. Third, a binary logistic regression model was used to analyze the associations between total fluid intake > 150 ml/kg/d, parenteral amino acid intake > 3.5 g/kg/d, total energy intake > 100 kcal/kg/d during the first week, feeding type, total enteral nutrition day, gestational age, birth weight, hsPDA and the occurrence of BPD.

All the analyses were conducted using SPSS software version 20.0. *p* < 0.05 indicated statistical significance.

## Results

### Demographic characteristics and primary outcome

**Fig. 1:**
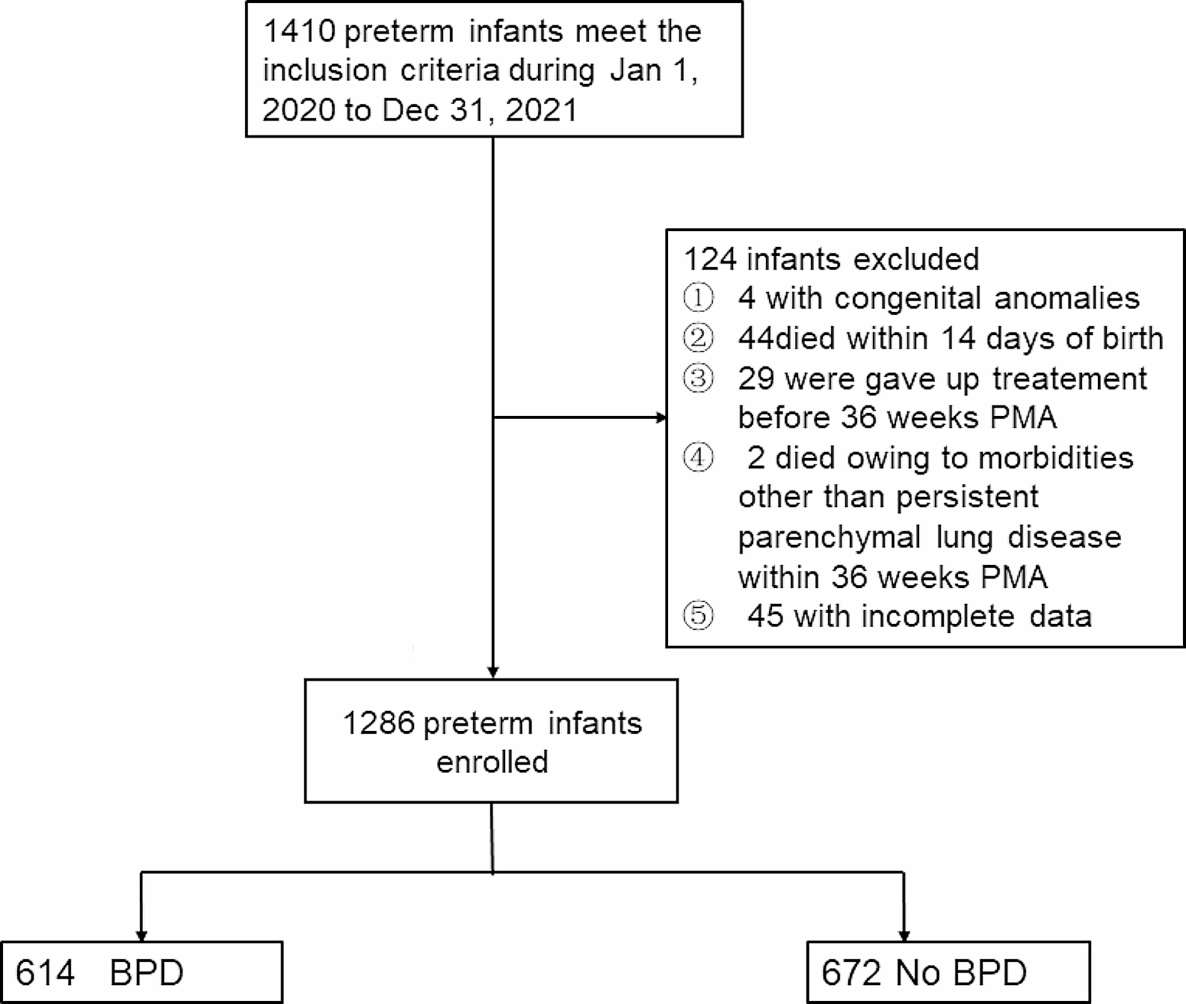
Flow diagram of enrolled participants

A total of 1410 preterm infants met the inclusion criteria between January 1, 2020, and December 31, 2021. Of these, 124 were excluded according to the exclusion criteria; therefore, 1286 infants were ultimately enrolled in the study. There were 614 preterm infants diagnosed with BPD, and the incidence of BPD in the study population was 47.7% (Fig. 1). Six infants (0.47%) who died before 36 PMA were diagnosed with grade IIIA BPD. Consequently, 345 (56.2%), 174 (28.3%), and 89 (14.5%) infants were diagnosed with grade I, II, and III BPD, respectively. Table 2 shows the demographic and clinical characteristics of infants with BPD and without BPD.

**Table 2:** Clinical and demographic characteristics of preterm infants with or without BPD*P < 0.05

	BPD I group 345 (56.2)	BPD II group 174 (28.3)	BPD III group 89 (14.5)	Non-BPD group 672 (52.3)	Z/χ^2^	*P* value
GA, weeks, median (IQR)	29 (27,30)	28 (27,29)	28 (27,29.3)	29 (28.0,30.0)	87.324	< 0.001^*^
BW, grams, median (IQR)	1185 (950,1463)	1070 (908,1293)	1045 (888,1243)	1340 (1100,1530)	112.246	< 0.001^*^
Male sex, n (%)	205 (59.4)	96 (55.2)	54 (60.7)	383 (57.1)	1.302	0.729
Apgar score at 5 min, score, n (%)					43.417	< 0.001^*^
<7	56 (16.2)	35 (20.1)	31 (34.8)	69 (10.3)		
≥7	289 (83.8)	139 (79.9)	58 (65.2)	603 (89.7)		
Maternal diabetes, N (%)	67 (19.4)	38 (21.8)	17 (19.1)	139 (20.7)	0.552	0.907
Maternal hypertension, N (%)	90 (26.1)	44 (25.3)	16 (18.0)	144 (21.4)	4.579	0.205
Assisted reproduction, N (%)	65 (19.0)	56 (32.2)	23 (25.8)	150 (22.4)	11.915	0.008^*^
PROM > 18 h,N (%)	87 (25.2)	35 (20.1)	17 (19.1)	183 (27.2)	5.621	0.132
IUGR, N (%)	25 (7.2)	15 (8.6)	11 (12.4)	47 (7.0)	3.512	0.319
Respiratory distress score at admission, score					59.890	< 0.001^*^
5–8, N (%)	311 (90.1)	150 (86.2)	72 (80.9)	658 (97.8)		
> 8, N (%)	34 (9.9)	24 (13.8)	17 (19.1)	15 (2.2)		
Surfactant, N (%)	272 (78.8)	160 (92)	83 (93.3)	414 (61.6)	99.386	< 0.001^*^
Mechanical ventilation, N (%)	201 (58.3)	139 (79.9)	76 (85.4)	281 (41.8)	125.018	< 0.001^*^
Ventilator days, ds, median (IQR)	6.0 (3.0,13.4)	9.6 (4.0,22.0)	21.3 (8.0,38.1)	3.0 (1.2,7.3)	241.258	< 0.001^*^
hsPDA, N (%)	106 (30.7)	59 (33.9)	38 (42.7)	115 (17.1)	50.748	< 0.001^*^
NEC(Stage ≥ 2), N (%)	38 (11.0)	33 (19.0)	20 (22.5)	41 (6.1)	41.383	< 0.001^*^
LOS, ds, median (IQR)	56 (44,73)	68 (54,86)	78 (60,106)	46 (35,59)	295.460	< 0.001^*^

BPD, bronchopulmonary dysplasia; GA, gestational age; BW, birth weight; PPROM, preterm premature rupture of membranes; IUGR, intrauterine growth retardation; hsPDA, hemodynamically significant patent ductus arteriosus; NEC, necrotizing enterocolitis; LOS, length of stay; IQR, interquartile range; SD, standard deviation

As expected, infants with BPD had a lower gestational age and birth weight (*P* < 0.001, *P* < 0.001). Compared to infants without BPD, those with BPD had lower Apgar scores at 5 min. Additionally, hsPDA and NEC were more common among infants with BPD (*P* < 0.001, *P* < 0.001). Moreover, infants with BPD had higher respiratory distress scores, more ventilator days, longer hospital stays, and more frequent ventilation use (Table 2). There was no significant difference in maternal history of hypertension or diabetes between infants with BPD and those without BPD. Clinical and demographic characteristics were compared between the BPD subgroup and the non-BPD subgroup and are presented in Supplemental Table 1.

### Patterns of nutritional attention

The total energy proportion reaching 100 kcal/kg/d during the first week was lower in the BPD subgroup than in the non-BPD subgroup (*P* < 0.001) and was lowest in the grade III BPD subgroup. The weight recovery days were significantly longer in the BPD subgroup, especially among those with grade III BPD (*P* = 0.024). Our study also focused on parenteral protein intake during the first week. The proportions of patients with parenteral AA > 3.5 gram/kg/d differed among the BPD subgroup and the non-BPD subgroup (*P* < 0.001). The percentages of patients in the BPD I, II and III groups were 62.9%, 71.3% and 52.8%, respectively, which were greater than those in the non-BPD group (Table 3).

**Table 3:** Nutritional status during the first week or at discharge comparisons between infants with and without BPD

	BPD I 345 (56.2)	BPD II 174 (28.3)	BPD III 89 (14.5)	Non-BPD 672 (52.3)	Z/χ^2^	*P* value
Total fluid > 150 ml/kg.d during the first wk, N (%)	164 (47.5)	87 (50.0)	51 (57.3)	310 (46.1)	4.319	0.229
Total energy reached 100 kcal/kg.d during the first wk, N (%)	203 (58.8)	88 (50.6)	35 (39.3)	411 (61.2%)	19.389	< 0.001^*^
Weight loss after birth > 10%, N (%)	50 (14.5)	31 (17.8)	21 (23.6)	106 (15.8%)	4.737	0.192
D of Weight recovery to BW, day, median (IQR)	9 (7,11)	8 (7,11)	10 (8,12)	8 (7,11)	9.484	0.024^*^
EUGR at 36 wks (PMA), N (%)	87 (25.4)	61 (35.1)	48 (54.5)	127 (19.1)	62.082	< 0.001^*^
Parenteral protein reached 3.5 g/kg.d during the first wk, N (%)	217 (62.9)	124 (71.3)	47 (52.8)	345 (51.3)	28.773	< 0.001^*^
D of full enteral nutrition, d, median (IQR)	27 (21,42)	30 (21,44)	45 (29,55)	21 (13,32)	165.242	< 0.001^*^
Type of feed, N (%)					53.483	< 0.001^*^
Breast milk	54 (15.7)	36 (20.7)	17 (19.5)	157 (23.5)		
Formula	75 (21.8)	15 (8.6)	16 (18.4)	92 (13.8)		
Mixed feed	88 (25.6)	80 (46.0)	39 (44.8)	206 (30.8)		
Breast milk ≥ 50%	127 (36.9)	43 (24.7)	15 (17.2)	213 (31.9)		

*P < 0.05 BPD, bronchopulmonary dysplasia; WK, week; D, day; EUGR, extrauterine growth retardation; PMA, postmenstrual age; IQR, interquartile range

Additionally, PN and EN were compared in all infants. The median duration (IQR) for infants without BPD who achieved full EN was 21 (13,32) days, while the average durations for those with BPD grades I, II, and III were 27 (21,42), 30 (21,44), and 45 (29,55) days, respectively. A longer duration was needed to establish full EN for infants with BPD and for those with increased severity.

Among the preterm infants, 735 (57.2%) achieved parenteral AA > 3.5 g/kg/d during the first week, while 551 (42.8%) did not. The duration of full EN achieved was significantly longer in the > 3.5 g/kg/d group during the first week than in the group that did not (28 days vs. 24 days, *P* < 0.001).

**Fig. 2:**
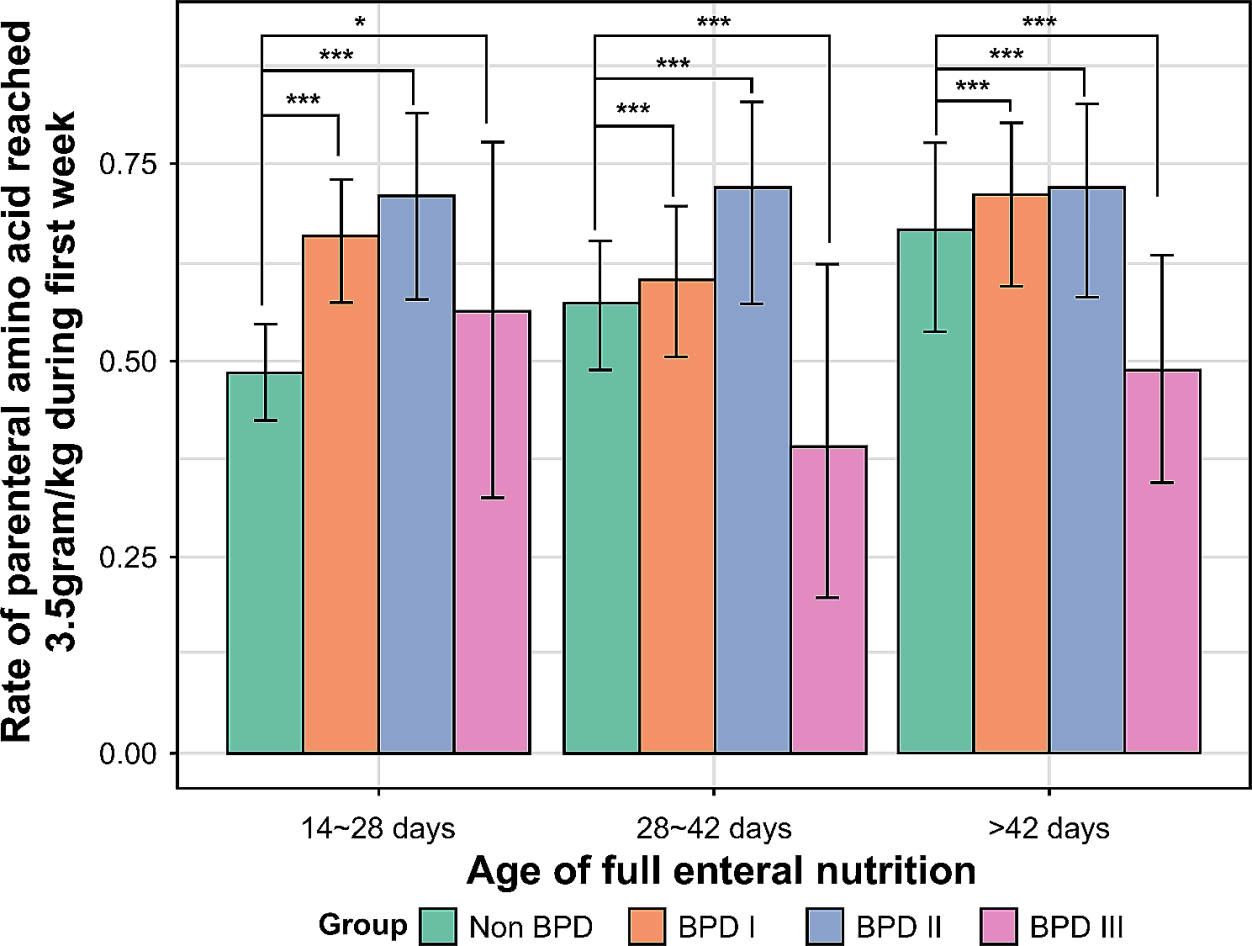
The parenteral amino acid concentration > 3.5 g/kg during the first week in the non-BPD and BPD subgroups

Additionally, infants were divided into groups according to the day at which full EN was reached: 14–28 days, 28–42 days, and > 42 days. The prevalence of parenteral AA > 3.5 g/kg/d during the first week was compared among the non-BPD subgroup and three BPD subgroups. Regardless of the full EN exposure period, the percentage of infants with parenteral AA > 3.5 g/kg/d was much lower among infants without BPD than among those with BPD grade I or II. However, this rate was significantly lower among infants with grade III BPD than among those without BPD, as the duration of full EN was longer (Fig. 2).

Our data demonstrated that feeding type differed among infants with and without BPD. The proportion of exclusively breast-milk-fed infants was 23.5% in the non-BPD group, which was the highest (*P* < 0.001).

The prevalence of IUGR did not significantly differ between infants with BPD and those without BPD; however, the prevalence of EUGR at 36 weeks PMA was significantly greater in the BPD subgroup than in the non-BPD subgroup. Moreover, the incidence of EUGR increased with BPD severity and was 25.4%, 35.1%, and 54.5% in the BPD I, II, and III; groups, respectively (Table 3).

Conversely, the proportion of infants exceeding 150 ml/kg/d fluid intake was similar among infants with and without BPD. Similarly, weight loss was not significantly different among infants. The results of the comparison of nutritional data between each BPD subgroup and the non-BPD subgroup are presented in Supplemental Table 2.

### Nutrition predictor variables for BPD development and secondary outcomes

Variables that were significantly different (at a level of *p* < 0.05) were used as predictors of BPD severity according to the multivariate logistic regression model. These variables included birthweight, gestational age, hsPDA, day of weight recovery to BW, day of full enteral nutrition, total fluid > 150 ml/kg/d during the first week, parenteral protein > 3.5 g/kg/d during the first week, total energy reaching 100 kcal/kg/d during the first week, feeding type, and EUGR at 36 weeks (PMA). The following six factors were associated with BPD according to odds ratios (ORs):

The inclusion criteria for participants were as follows: birth weight (OR 0.99, 95% CI 0.99–0.99; *p* = 0.039); day of full enteral nutrition (OR 1.03, 95% CI 1.02–1.04; *p* < 0.001); parenteral protein > 3.5 g/kg/d during the first week (OR 1.65, 95% CI 1.25–2.17; *p* < 0.001); feeding type (formula: OR 3.48, 95% CI 2.21–5.49; *p* < 0.001; mixed feed: OR 1.92, 95% CI 1.36–2.70; *p* < 0.001; breast milk as reference); hsPDA (OR 1.98, 95% CI 1.44–2.73; *p* < 0.001); and EUGR at 36 weeks (OR 1.40, 95% CI 1.02–1.91; *p* = 0.035) (Table 4).

**Table 4:** Multivariate logistic regression analysis of the determinants of BPD

	Z	P	OR (95%CI)
GA	-1.63	0.104	0.91 (0.81–1.02)
BW	-2.07	0.039*	0.99 (0.99–0.99)
Day of Weight recover to BW	0.42	0.676	1.01 (0.97–1.04)
D of full enteral nutrition	6.50	< 0.001*	1.03 (1.02–1.04)
Total fluid > 150 ml/kg.d during the first wk	0.56	0.573	1.08 (0.82–1.43)
Parenteral protein reached 3.5 g/kg.d during the first wk	3.52	< 0.001*	1.65 (1.25–2.17)
Total energy reached 100 kcal/kg.d during the first wk	0.10	0.920	1.01 (0.77–1.34)
Feeding type			
Breast milk			1.00 (Reference)
Formula	5.37	< 0.001*	3.48 (2.21–5.49)
Mixed feed	3.74	< 0.001*	1.92 (1.36–2.70)
hsPDA	4.18	< 0.001*	1.98 (1.44–2.73)
EUGR at 36 wks (PMA)	2.11	0.035*	1.40 (1.02–1.91)

*P < 0.05 BPD, bronchopulmonary dysplasia; WK, week; D, day; EUGR, extrauterine growth retardation; PMA, postmenstrual age; hsPDA, hemodynamically significant patent ductus arteriosus; BW, birth weight; GA, gestational age; D, days

## Discussion

Our results demonstrated that nutritional support was associated with the progression of BPD in infants aged < 32 weeks with respiratory distress. A duration of full enteral nutrition was related to BPD occurrence. These findings are consistent with those of previous studies. Retrospective studies by Wemhöner et al. [13] and Uberos et al. [14] revealed that preterm infants who developed BPD received less enteral feed. According to a recent study by Lin et al. [15], a 10% increase in the enteral feeding/total fluid intake ratio dramatically decreased the incidence of BPD by 55.6% during the second week.

Regarding the feed type, compared with an exclusive formula diet and mixed feed, our study suggested that exclusive breastfeeding protected against BPD among very preterm infants. A range of nutrients and physiologically active compounds found in human milk may be crucial for preventing BPD, as they assist neonates in developing their innate immune and antibacterial capabilities [16, 17]. Breast milk contains high tocopherol, carotene, and other antioxidant levels that help preterm infants with oxidative stress and lower their BPD development risk [18, 19].

The relationship between breastfeeding and BPD has also been largely investigated. A systematic review [20] of 17 cohorts and five randomized control trials involving 8661 preterm infants revealed that both exclusive and partial human milk feeding appeared to be associated with a lower BPD risk in preterm infants. A meta-analysis revealed that maternal milk (MOM) may reduce the incidence of BPD [21]; similarly, donor human milk (DHM) has a similar effect as formula [22]. Recent research has indicated that breast milk is not only associated with short-term complications such as BPD but also improves lung function in the long-term. Lavizzari A et al. reported that preterm infants born to ELBW mothers with higher consumption of HMs had significantly less airway obstruction during the first 2 years [23].

Our data showed that hsPDA was associated with an increased risk of BPD, similar to longer full enteral nutrition. hsPDA was a common complication in preterm infants born at < 32 weeks of gestation. A large left-to-right PDA shunt significantly increases the risk of developing BPD or death, as shown in previous studies [24]. When hsPDA is present, the blood flow “steal” from the descending aorta to pulmonary arteries may exceed the physiological compensatory mechanisms aimed at increasing left cardiac output, with subsequent reductions in end-organ perfusion, such as through the gastrointestinal (GI) tract. The development of gastrointestinal complications in preterm infants with hsPDA is a common fear among neonatologists and represents a major challenge for the management of enteral feeds in this delicate population [25]. The presence of hsPDA was associated with a significant delay in enteral feeding introduction and a significantly greater frequency of full enteral feeding (OR 2.80, 95% CI 1.35–5.81; *p* = 0.006) [26]. The main pharmacological agents used for PDA closure, such as indomethacin, ibuprofen, and paracetamol, also have possible adverse effects on the GI tract, which may influence enteral feeding [27]. Likewise, for preterm infants receiving PDA ligation, full enteral feeding achievement was greatly delayed [28]. Thus, hsPDA may influence the establishment of enteral nutrition, and a longer duration is needed to achieve full enteral nutrition. However, hsPAD and longer full enteral nutrition increased the risk of BPD development.

As nutritional support during the first week of life for very preterm infants, both enteral and parenteral nutrition are crucial and interrelated. Our data showed that infants without BPD reached full EN earlier and that their rate of reaching parenteral protein was lower during the first week. In contrast, the percentage of infants with parenteral protein > 3.5 g/kg/d was greater in infants with BPD due to delayed enteral nutrition. Thus, a parenteral protein concentration > 3.5 g/kg/d was associated with BPD development and was the reason for the longer duration of full enteral nutrition. It was recommended that the amino acid supply in parenteral nutrition start on the first postnatal day at least 1.5 g/kg/d to achieve an anabolic state and between 2.5 g/kg/d and 3.5 g/kg/d and should be accompanied by nonprotein intake > 65 kcal/kg/d and adequate micronutrient intake beginning on day 2 of life according to the ESPGHAN/ESPEN/ESPR guidelines. It has also been shown that parenteral amino acid intake above 3.5 g/kg/d should be administered only as part of clinical trials [29]. In addition to the amount of parenteral protein, it is also necessary to consider the nonprotein energy and the energy–nitrogen ratio. This topic requires further study.

Our results demonstrated that infants with BPD had lower energy intake during the first week, increased EUGR incidence at 36 weeks PMA, and prolonged days for birth weight recovery. Adequate nutrition is required for somatic and lung growth promotion. The effects of postnatal malnutrition on lung development have been studied in animal models. Early postnatal nutritional restriction impairs alveolarization and impacts the bronchiolar epithelium, therefore decreasing epithelial cell division and Clara cell conversion to ciliated cells [30, 31]. According to other animal studies, malnutrition worsens the detrimental effects of hyperoxia on lung alveolarization and extracellular matrix deposition [32, 33]. Consistent with our findings, other studies [34] have reported lower caloric intake among infants with BPD during the first two weeks. However, unlike these studies [14, 34], our study did not confirm that early lower caloric intake after birth was a risk factor for BPD occurrence.

The main strength of our study was that it was the first prospective multicenter observational study across the Chinese mainland with a large sample size exploring the role of early nutritional status in BPD. Nonetheless, our study has several limitations. First, we did not collect specific values for certain nutritional metrics but rather collected information on whether a certain indicator was achieved, such as a total volume > 150 ml/kg/d and total energy > 100 kcal/kg/d. Second, we did not collect data on trace elements, electrolytes, lipids or carbohydrates, which are also important for nutritional support. Despite these limitations, this study explored the outcomes and risk factors associated with early nutritional support for pregnant BPD infants aged < 32 weeks with respiratory distress in China. These findings could lead to further BPD treatment and management.

In conclusion, a longer duration to achieve full enteral nutrition in very preterm infants was associated with increased BPD development. Breastfeeding was demonstrated to have a protective effect against BPD. Early and rapidly progressive EN and breastfeeding should be promoted in very preterm infants.

### Electronic supplementary material

Below is the link to the electronic supplementary material.

Supplementary Material 1

## Data Availability

The datasets used and/or analyzed during the current study are available from the corresponding author upon reasonable request.

## Abbreviations

AA: Amino acid
BPD: Bronchopulmonary dysplasia
BW: Birth weight
ChiCTR: Chinese Clinical Trial Registry
EN: Enteral nutrition
EUGR: Extrauterine growth retardation
GA: Gestational age
hsPDA: Hemodynamically significant patent ductus arteriosus
IQRs: Interquartile ranges
IUGR: Intrauterine growth retardation
LA/AO: Left atrial inner diameter/aortic root
NEC: Necrotizing enterocolitis
NICHD: National Institute of Child Health and Human Development
NICUs: Neonatal intensive care units
PMA: Postmenstrual age
PN: Parenteral nutrition
