# Supplementary material for: Nutritional support during the first week for infants with bronchopulmonary dysplasia and respiratory distress: a multicenter cohort study in China
Source: BMC Pediatr. 2024 Apr 3;24:238. doi: 10.1186/s12887-024-04675-5 (PMC10988891; doi:10.1186/s12887-024-04675-5)
Supplement: Supplementary file 1 — Supplementary Material 1 [file 12887_2024_4675_MOESM1_ESM.docx]

**Supplementary table 1 Clinical and demographic characteristics for preterm infants with or without BPD **P* < 0.05(post hoc analysis for subgroups of BPD vs non-BPD group)**

|  | *P^#^* | *P_a_* | *P_b_* | *P_c_* |
| --- | --- | --- | --- | --- |
| GA, weeks, median (IQR) | <0.001^*^ | <0.001* | <0.001* | <0.001* |
| BW, grams, median (IQR) | <0.001^*^ | <0.001^*^ | <0.001^*^ | <0.001^*^ |
| Male sex, n (%) | 0.729 | >0.05 | >0.05 | >0.05 |
| Apgar score at 5 min, score,  n (%) | <0.001^*^ | <0.05* | <0.05* | <0.05* |
| <7 |  |  |  |  |
| ≥7 |  |  |  |  |
| Maternal diabetes, N (%) | 0.907 | >0.05 | >0.05 | >0.05 |
| Maternal hypertension, N (%) | 0.205 | >0.05 | >0.05 | >0.05 |
| Assisted reproduction, N (%) | 0.008^*^ | >0.05 | <0.05* | <0.05* |
| PROM > 18 h,N (%) | 0.132 | >0.05 | >0.05 | >0.05 |
| IUGR, N (%) | 0.319 | >0.05 | >0.05 | >0.05 |
| respiratory distress score at admission, score | <0.001^*^ | <0.05* | <0.05* | <0.05* |
| 5—8,N (%) |  |  |  |  |
| > 8, N (%) |  |  |  |  |
| Surfactant, N (%) | <0.001^*^ | <0.05* | <0.05* | <0.05* |
| Mechanical ventilation, N(%) | <0.001^*^ | <0.05* | <0.05* | <0.05^*^ |
| Ventilator days, ds, median (IQR) | <0.001^*^ | <0.001* | <0.001* | <0.001* |
| hsPDA, N (%) | <0.001^*^ | <0.05* | <0.05* | <0.05* |
| NEC(Stage≥2), N (%) | <0.001^*^ | <0.05* | <0.05* | <0.05* |
| LOS, ds, median (IQR) | <0.001^*^ | <0.001^*^ | <0.001^*^ | <0.001^*^ |

#For comparisons of infants without and with BPD among different severity, Kruskal–Wallis test and chi square were adopted, as appropriate. a For comparisons of infants non-BPD and with BPD Ⅰ group, one-way ANOVA test and chi square were adopted, as appropriate. b For comparisons of infants non-BPD and with BPD Ⅱ group, one-way ANOVA test and chi square were adopted, as appropriate. c For comparisons of infants non-BPD and with BPD Ⅲ group, one-way ANOVA test and chi square were adopted, as appropriate.

BPD, bronchopulmonary dysplasia; GA, gestational age; BW, birth weight; PPROM, preterm premature rupture of membranes; IUGR, intrauterine growth retardation; hsPDA, hemodynamically significant patent ductus arteriosus; NEC, necrotizing enterocolitis; LOS, length of stay; IQR, interquartile range; SD, standard deviation.

**Supplementary table 2 Nutrition status during the first week or at discharge comparisons between infants with and without BPD**

|  | *P^#^* | *P_a_* | *P_b_* | *P_c_* |
| --- | --- | --- | --- | --- |
| Total fluid > 150 ml/kg.d during the first wk, N (%) | 0.229 | <0.05* | <0.05* | <0.05* |
| Total energy reached 100 kcal/kg.d during the first wk, N (%) | <0.001^*^ | <0.05* | <0.05* | <0.05* |
| Weight loss after birth > 10%, N (%) | 0.192 | <0.05* | >0.05 | <0.05* |
| D of Weight recovery to BW, day, median (IQR) | 0.024^*^ | >0.05 | >0.05 | 0.17* |
| EUGR at 36 wks (PMA), N (%) | <0.001^*^ | <0.05* | <0.05* | <0.05* |
| Parenteral protein > 3.5 g/kg.d during the first wk, N (%) | <0.001^*^ | <0.05* | <0.05* | <0.05* |
| D of full enteral nutrition, d, median (IQR) | <0.001^*^ | <0.001^*^ | <0.001^*^ | <0.001^*^ |
| Type of feed, N (%) | <0.001^*^ | <0.05* | <0.05* | <0.05* |
| Breast milk |  |  |  |  |
| Formula |  |  |  |  |
| Mixed feed |  |  |  |  |
| Breast milk ≥ 50% |  |  |  |  |

#For comparisons of infants without and with BPD among different severity, Kruskal–Wallis test and chi square were adopted, as appropriate. a For comparisons of infants non-BPD and with BPD Ⅰ group, one-way ANOVA test and chi square were adopted, as appropriate. b For comparisons of infants non-BPD and with BPD Ⅱ group, one-way ANOVA test and chi square were adopted, as appropriate. c For comparisons of infants non-BPD and with BPD Ⅲ group, one-way ANOVA test and chi square were adopted, as appropriate. *P < 0.05

BPD, bronchopulmonary dysplasia; WK, week; D, day; EUGR, extrauterine growth retardation; PMA, postmenstrual age; IQR, interquartile range.
